# Epidemiological, clinical and laboratorial profile of renal amyloidosis: a 12-year retrospective study of 37 cases

**DOI:** 10.12860/jnp.2015.02

**Published:** 2015-01-01

**Authors:** Elissa Oliveira da Fonseca, Porphirio Jose Soares Filho, Licinio Esmeraldo da Silva, Maria Lucia Ribeiro Caldas

**Affiliations:** ^1^Hospital Universitario Antonio Pedro, Universidade Federal Fluminense, Rio de Janeiro, Brazil

**Keywords:** Amyloidosis, Proteinuria, Kidney biopsy, Nephrotic syndrome

## Abstract

*Background:* Renal amyloidosis is one of the main differential diagnoses in the investigation of nephrotic proteinuria in adults, especially elderly patients.

*Objectives:* The aim of this article is to contribute to international research with epidemiologic data of renal amyloidosis, given the lack of uniformity described in the literature.

*Patients and Methods:* A retrospective study of 37 cases of renal amyloidosis diagnosed by kidney biopsy, between 2000 and 2011, considering epidemiological, clinical and laboratory data.

*Results:* Subjects aged between 32 and 80 years. Of the 37 cases, 21 (56.8%) were diagnosed as non-light chain (non-AL) renal amyloidosis and 16 (43.2%) as light chain amyloidosis (AL). There was seen an increase in number of both AL and non-AL cases, with a slight predominance in non-AL. The mean 24-hour proteinuria was 5839.0 mg/day. Hematuria was present in 75% of patients. Hypertension was reported in 34% of patients. Acute renal failure, occurred in about 10% of patients, and chronic loss of renal function was present in about 5% at diagnosis.

*Conclusions:* Renal amyloidosis is a disease of increasing incidence. The forms of clinical presentation proved to be variable, but the presence of proteinuria or nephrotic syndrome in elderly patients should always prompt the suspicion of renal amyloidosis and is a formal indication of renal biopsy.

**Implication for health policy/practice/research/medical education:**

Renal amyloidosis is a disease of increasing incidence. The forms of clinical presentation proved to be variable, but the presence of proteinuria or nephrotic syndrome in elderly patients should always prompt the suspicion of renal amyloidosis and is a formal indication of renal biopsy.

## 1. Background

Amyloidosis is a group of disorders resulting from extracellular tissue deposition of autologous proteins with abnormal folding in non-parallel arrangement of trans-beta-sheet-forming non-branched and linear long fibrils (1,2). These proteins are known as amyloid and even with multiple compositions share the same physical properties and histological staining by optical microscopy, showing specific affinity for Congo red (CR) and similar ultrastructural analysis of amyloid fibrils (3).

The kidneys are one of the most frequently affected organs, and renal amyloidosis is one of the main differential diagnoses in the investigation of nephrotic proteinuria in adults, especially elderly patients (4). Classification of the amyloidosis is based on the precursor protein that forms the amyloid fibrils and the distribution of amyloid deposition as either systemic or localized. The major types of systemic amyloidosis are amyloid A (AA), light chain associated (AL) and the hereditary amyloidosis (5). AA amyloidosis is associated with longstanding chronic inflammatory processes like rheumatoid arthritis, chronic osteomyelitis, inflammatory bowel disease, familial Mediterranean fever and tuberculosis (6). AL amyloidosis can be caused by neoplastic expansion of plasma cell population synthesizing amyloidogenic light chains (7). In the hereditary type, an inherited gene mutation renders a protein amyloidogenic. Among them, we can mention transthyretin, fibrinogen Aα, lysozyme, apolipoprotein AI, apolipoprotein AII, gelsolin, and cystatin (5). A novel renal amyloid protein was recently described, the leukocytic chemotactic factor 2 (LECT2), with hereditary and/or environmental factors seemed to contribute to disease pathogenesis (8,9).

The diagnosis is usually made at an advanced stage of the disease, bringing difficulty in achieving adequate therapeutic responses leading to progression to chronic renal failure (10). The literature reveals no uniform epidemiological data on this disease (11) and still no clear associations between the extent of amyloid deposits identified on renal biopsy and clinical manifestations were demonstrated (12). A recent study on the prevalence of biopsy-proven renal disease indicates amyloidosis as the second most common secondary glomerular disease, outnumbered only by lupus nephritis (13). Another study demonstrates that renal amyloidosis is the major biopsy diagnosis in elderly patients over 85 years (14). In Brazil, a recent study of 9617 renal biopsies rate amyloidosis as the second main glomerulonephritis associated with metabolic diseases (31.3%) and one of the most frequent diagnoses in the elderly [membranous nephropathy, 13.1%; focal segmental glomerulosclerosis (FSGS), 12.1%; amyloidosis, 9.8%; diabetic nephropathy, 7.5% and benign and malign nephroangiosclerosis, 6.1%] (11).

## 2. Objectives

The literature reveals a distinct lack of uniformity in the collection of epidemiological data on renal amyloidosis. The aim of this article is to characterize the features of clinical and laboratory presentation of renal amyloidosis, through data analysis from clinical protocol, relating with the main types of renal amyloid by histological techniques [immunofluorescence (IF), immunohistochemistry and CR staining]. Given the paucity of studies from developing countries and the lack of uniformity described in the literature on this subject, we believe, based also on the geographic diversity, that this study can enrich the understanding of this disease.

## 3. Patients and Methods

### 3.1. Patients

We present a retrospective study of renal amyloidosis diagnosed by kidney biopsy between 2000 and 2011, taking into account epidemiological, clinical and laboratory data obtained by protocol to request the renal biopsy.

### 3.2. Histologic data

The material corresponds to 37 reports of native kidney biopsies, kindly provided by the nephropathology consultant of the Bio Neo Laboratory, Rio de Janeiro. These biopsies represent all the cases diagnosed as renal amyloidosis during the period studied at the mentioned center. The reported biopsies were fixed with 10% buffered formalin, processed according to the routine and embedded in paraffin. Serial sections were performed with 3 μm thick on 5 levels stained with hematoxylin and eosin, Schiff periodic acid, Masson’s trichrome and silver methenamine stains. Additional cuts of 7 μm thick were stained with Highman’s Congo red and analyzed in brightfield and polarized light, required to establish the diagnosis of amyloidosis. There were also samples for IF, looking for AL amyloidosis and differentials. The material was carried in Michel solution, washed in PBS, pH 7.4, submitted to liquid nitrogen, embedded in Tissue Tek^®^, cut in a cryostat at -20 °C in 5 μm and incubated with antibodies (rabbit sera; polyclonal anti-human conjugated to fluorescein isothiocyanate) supplied by DAKO^®^ (IgA, IgG, IgM, C3, C1q, C4, fibrinogen, light chain kappa and lambda light chain). Only 2 cases had remaining paraffin embedded tissue for undergoing additional sections for immunohistochemistry, looking for AA amyloidosis (monoclonal mouse antibody to human amyloid A component, DBS, clone mc1, catalog’s number Mob 003, dilution titer of 1:25 - 1:150), showing positivity in the glomeruli and vessels. Therefore, we preferred to name “non-AL” all negative cases for light chains by IF technique analysis, as others subtypes can be involved, for example, hereditary amyloidosis.

Clinical, laboratory and epidemiological data were recorded at the time of biopsy and were subsequently evaluated together with the histopathologic findings (light microscopy, IF and CR). Among them, we can mention, age, sex, race, indication for renal biopsy, hypertension, edema, arthritis/arthralgia, neuropathies, associated diseases, vomiting, skin lesions, 24-hour proteinuria, virology (HBsAg, HCV, HIV) and serology (ANA, anti-DNA, C3 and C4).

### 3.3. Ethical issues

1) The research followed the tenets of the Declaration of Helsinki; 2) informed consent was obtained; and 3) the research was approved by the institutional review board.

### 3.4. Statistical analysis

Age, gender, clinical and laboratorial values were considered for comparisons with literature. The data were analyzed statistically with the SPSS^®^ 10.0 and Microsoft Excel program, for calculation of mean values, standard deviation, maximum and minimum, median and interquartile ranges. Crosstables and graphics also helped the analysis.

## 4. Results

**Figure 1:**
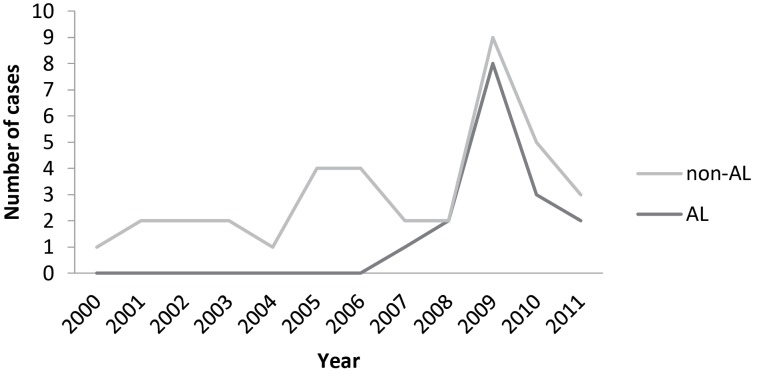
Distribution of cases per year

**Figure 2:**
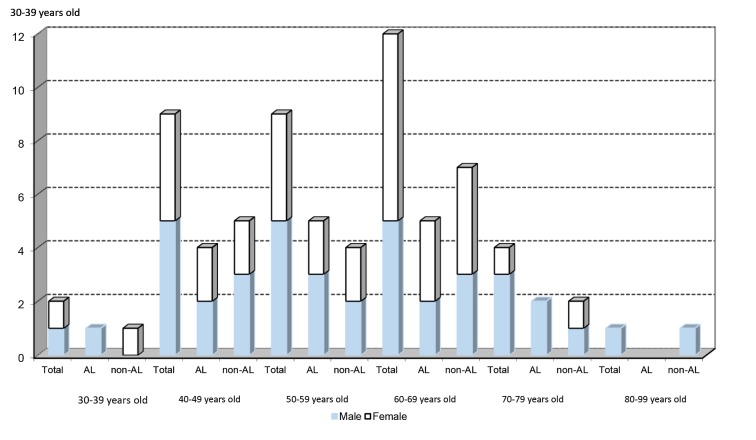
Distribution of cases per year

From 2000 to 2011, 37 cases of renal amyloidosis were diagnosed by needle biopsy from a total of 1609 biopsies, corresponding to 2,3%. Eighteen of the selected patients were females and 19 males, with a larger number of cases in recent years, as seen in Figure 1. Of the 37 cases, 21 (56.8%) were diagnosed as non-light chain (non-AL) renal amyloidosis and 16 (43.2%) as light chain (AL) amyloidosis. There was an increase in the number of both AL and non-AL cases, with a slight predominance in non-AL. The age ranged between 32 and 80 years old, mean 57 years, with 62.2% of cases over 50 years. The average age among non-AL cases was 57.9 years and in AL cases it was 56.6 years. Frequency of different types of amyloidosis according to age distribution is reported in Figure 2: 2 patients aged between 30 and 39 years; 9 between 40 and 49 years; 9 between 50 and 59 years; 12 between 60 and 69 years, 4 between 70 and 79 years; and 1 patient with 80 years. There were 12 female patients with non-AL, 10 male with non-AL, 6 female with AL and 9 male with AL amyloidosis. As far as gender is concerned, females exhibited a similar predisposition when compared with males in our study (48.6% of the cases were from female patients and 51.4% from males).

Twelve patients showed an increase of creatinine at the time of biopsy, eight of these with AL amyloidosis. Hypertension (levels above 140/90 mmHg) was seen in 14 (37.8%) of our patients, 50% of them with global glomerulosclerosis by light microscopy (ranging from 3.4% to 38.9% of the glomeruli). The predominant sites of amyloid deposition were glomeruli (97% of the cases), frequently with associated tubulointerstitial and vascular deposits (21% and 57%, respectively). Sixty seven percent of the biopsies had few focal areas of interstitial fibrosis and 12% has multiple foci of fibrosis. Fifteen percent had no interstitial fibrosis. One of the 37 patients showed familial history for renal disease (not specified) corresponding to a 51 years old female, with light chain positivity in the glomeruli by IF, diagnosed as AL amyloidosis.

Laboratory tests showed 24-hour urine protein excretion average of 5839 mg/day (range: 979 mg/day to 17900 mg/day). Only 10.8% of the patients had proteinuria of between 1 g/day and 3,5 g/day and 54% had nephrotic range proteinuria. Proteinuria lower than 1 g/day was present in 2.7% of the patients. Hypoalbuminemia was found in 2 (5.4%) patients. Hypercholesterolemia was seen in 6 patients. Nephrotic syndrome was reported in 9 of the 37 studied patients (24%) and 5 patients had only edema with no associated nephrotic syndrome. Hematuria was detected in 75% of patients. Acute renal failure, was reported in 10% of patients, and chronic loss of renal function was present in 5% at diagnosis. None of the fifteen tested patients were positive for HIV or hepatitis B or C viruses. The four cases for which kidney radiology had been provided showed normal size and appearance.

Fourteen patients presented with hypertension (8 non-AL, 6 AL), 2 with heart failure (1 non-AL, 1 AL), 2 with positive ANA (non-AL), and 6 with hypercholesterolemia (3 non-AL, 3 AL). Only one patient had urine protein electrophoresis with no Bence Jones proteins found at the time of the biopsy. Neither atypical intratubular casts were found in the biopsies (the eventual casts described by light microscopy were proteinaceous) and there was also no light chain casts by IF. There were also no diabetic patients. Demographic, clinical and biochemical characteristics of the studied patients are presented in Table 1.

**Table 1:** Demographic, clinical and biochemical characteristics of the studied patients

**Parameters**	**Non-AL amyloidosis**	**AL amyloidosis**
Numbers of patients	21	16
Age (years)	57.9 ± 12.7	56.6 ± 11
Sex		
Female	11 (29.7%)	7 (18.9%)
Male	10 (27.1%)	9 (24.3%)
Proteinuria	5.9 ± 4.3	5.8 ± 3.6
Hematuria	5 (13,5%)	4 (10.8%)
Anti-HCV	negative	negative
Anti-HBV	negative	negative
Anti-HIV	negative	negative
Creatinine	3.4 ± 3.4	1.9 ± 1.3
Edema	5 (13,5%)	2 (5.4%)
Nephrotic syndrome	4 (10.8%)	5 (13,5%)
Urinary Bence Jones protein	negative	negative
Arthritis/arthralgia	2 (5.4%)	-
Skin lesions	1 (2.7%)	-
Oliguria/anuria	1 (2.7%)	1 (2.7%)
AKI (acute kidney injury)	2 (5.4%)	2 (5.4%)
CKD (chronic kidney disease)	1 (2.7%)	1 (2.7%)
Hypoalbuminemia	1 (2.7%)	1 (2.7%)

## 5. Discussion

Recently, an increase in the number of cases of renal amyloidosis has been observed by Tsai *et al*. (12), which refer this trend as a consequence of obscure underlying mechanisms, but may probably be partly due to the aging of the general population which results in more people at high risk age for developing amyloidosis. They also hypothesized that aging might still be an important contributing factor to the increasing incidence in AL amyloidosis. Most non-AL amyloidosis cases are due to chronic inflammation, which is not age-dependent (12). In our study, there was seen an increase in the numbers of both AL and non-AL cases, with a slight predominance in non-AL. von Hutten *et al*. interestingly have found that the absolute and relative prevalence of renal AA amyloidosis and AL amyloidosis did not show any significant tendencies toward a constant decrease or increase during their entire study period of seventeen years (4). Pettersson *et al*. (2) reported that the incidence of AL amyloidosis has been stable over the years. Bergesio *et al*. (6), in a retrospective study with Italian population, found similar median age on comparing non-AL and AL cases, with a wide range of distribution. In this referred study, female gender was prevalent in non-AL, while males were more prevalent in AL cases. In our study, the same trend was observed, with 12 female showing non-AL, 10 male non-AL, 6 female AL and 9 male AL amyloidosis. Bergesio *et al*. and Von Hutten *et al*. (4,6) reported a greater number of AL cases (63.5% and 53.7%), and this can be due to what the literature has shown about the prevalence of AL amyloidosis in developed countries (2). The Italian study (6) also showed that median age at diagnosis was 63 years (range 19 to 88), with no significant differences between males (median 63) and females (median 66). Females denoted a similar trend when compared with males in our study, as others authors have shown the same aspect (6,12). Nevertheless, Tsai *et al*. (12) noted in their cohort that the total number of male cases exceeded that of females and the incidence of AL amyloidosis appeared to be increasing in females. Key issues regarding whether females are becoming more susceptible to the development of AL amyloidosis remains uncertain in recent years, as it is a new information in the literature in need for further investigation (12).

In Bergesio *et al*., study, ten patients were on dialysis at the time of diagnosis, five of them were AL, and five non-AL amyloidosis (6). On progression, azotemia and renal failure as well as nephrogenic hypertension, usually only mild or moderate, will develop in patients with renal amyloidosis (2). Certain degree of renal function impairment can be associated combined with high levels of blood pressure (12), which is seen in 14 (37.8%) of our patients and was present in 12.8% of patients from a recent study from Cairo with 40 patients from 2003 to 2009 (15). Hypertension is described as an uncommon feature of amyloidosis, which can follow vascular involvement (15). Many cases of amyloidosis are characterized by kidney involvement, which is defined as 24-hour urinary protein, mostly albumin, exceeding 0.5 g. Laboratory tests showed 24-hour urine protein excretion average of 5839 mg/dL (range: 979 mg/dL to 17900 mg/dL) in our study population. According to other authors (4,12), proteinuria was above the nephrotic range (5.8 ± 3.9 g/day). Proteinuria was the most frequent laboratory finding reported by other authors, and usually has a variable spectrum, from asymptomatic to severe nephrotic syndrome with massive urine excretion rates as high as 20 to 30 g/d. If proteinuria exceeds 3 to 5 g per 24 hours, hypoalbuminemia ensues (2), and it was found in 2 (5.4%) patients.

Hypercholesterolemia is seen in 6 patients and, according to the literature, it often develops, caused by multiple mechanisms related to urinary loss of various proteins regulating cholesterol metabolism, and leading to impaired LDL clearance, increased cholesterol synthesis, diminished high-density lipoprotein (HDL)-mediated reverse cholesterol transport, and diminished cholesterol (bile acid) secretion (2). Hematuria was detected in 75% of patients, contradicting the literature that describes this as a rare finding (2).

Like other infiltrative diseases, amyloidosis can cause enlargement of the kidneys. However, in most patients, the kidneys seem to be of normal size by imaging studies, and the absence of enlarged kidneys should not decrease suspicion for the disease during diagnostic evaluation (5).

There was no clinical information about lymphoproliferative diseases and neither urinary Bence Jones proteins. Pettersson and Konttinen verified, in their study from 2010, that 15% of patients with AL amyloidosis fulfill the criteria for multiple myeloma. According to the same study, amyloidosis will develop in about 15% of patients with myeloma (2).

## 6. Conclusions

Renal amyloidosis is a disease of increasing incidence, with a greater number of non-AL cases and non gender differences in our cohort. This may partly be due to the prevalence of rare group of hereditary amyloidosis. Despite its increasing incidence, amyloidosis is still a rare disorder with a variable clinical presentation. Proteinuria with or without nephrotic syndrome is the most common clinical finding, and this finding in elderly patients should be a formal indication of renal biopsy as a diagnostic tool. Overall renal function seems to be preserved at the time of the diagnosis, in this selected group, and hypertension is not as uncommon as the literature referred. Epidemiologic data in literature reports is variable, depending on the population. Several conditions can be associated with AL or non-AL amyloidosis, and clinical information is essential for the correct diagnosis.

## Acknowledgements

Prof Carlos Alberto Basilio de Oliveira and Bio Neo Laboratory for contributing with essential material for the study.

## Conflict of interests

Authors declare no conflict of interests.

## Authors’ contributions

All authors wrote the paper equally.

## Funding/Support

None.
